# New Weekly Journal

**Published:** 1893-02

**Authors:** 


### NEW WEEKLY JOURNAL.

THE *Dental Tribune*, a newspaper for dentists, issued every Saturday, and edited by Dr. Louis Ottofy, Chicago, Ill., is another candidate for the interest of the profession.

The numbers already issued indicate a determined purpose to make it of value in a general sense. Its size does not warrant the expectation of its being a medium for scientific dental literature, and we do not understand this to be the intention. It will fill a place as a dental news journal, especially at the period of the Columbian Exhibition.

The numbers thus far have been mainly filled with interesting sketches of the men prominent in the organization of the World's Columbian Dental Congress.

The editor has the best wishes of this journal for a successful result as a reward for his earnest and courageous effort.

